# Deregulation of MUC4 in gastric adenocarcinoma: potential pathobiological implication in poorly differentiated non-signet ring cell type gastric cancer

**DOI:** 10.1038/sj.bjc.6604632

**Published:** 2008-09-09

**Authors:** S Senapati, P Chaturvedi, P Sharma, G Venkatraman, J L Meza, W El-Rifai, H K Roy, S K Batra

**Affiliations:** 1 Department of Biochemistry and Molecular Biology, University of Nebraska Medical Center, Omaha, NE 68198, USA; 2 Department of Pathology, Creighton University Medical Center, Omaha, NE 68131, USA; 3 Department of preventive and Societal Medicine, University of Nebraska Medical Center, Omaha, NE 68198, USA; 4 Department of Surgery, Vanderbilt University Medical Center, Nashville, Tennessee 37232, USA; 5 Northwestern University Feinberg School of Medicine, Division of Gastroenterology, Evanston Northwestern Healthcare, Evanston, IL 60201, USA

**Keywords:** MUC4, mucin, gastric adenocarcinoma, signet ring cell carcinoma

## Abstract

MUC4 is a large, heavily glycosylated transmembrane mucin, that is implicated in the pathogenesis of various types of cancers. To date, no extensive study has been done to check the expression and functional significance of MUC4 in different types of gastric adenocarcinomas. Here, we report the expression profile of MUC4 in gastric adenocarcinomas and its function in poorly differentiated gastric non-signet ring cell carcinoma (non-SRCC) type cells. Immunohistochemical analysis using tissue microarray (TMA) showed a significant difference in MUC4 expression between normal adjacent (*n*=45) and gastric adenocarcinoma (*n*=83; *P*<0.001). MUC4 expression was not associated with tumour type, stage or with the degree of differentiation. To gain further insight into the significance of MUC4 expression in gastric non-SRCC cells, MUC4 was ectopically expressed in AGS, a poorly differentiated gastric non-signet ring cell line. The MUC4 overexpressing cells (AGS-MUC4) showed a significant increase (*P*<0.005) in cell motility and a decrease in cellular aggregation as compared with the vector-transfected cells. Furthermore, *in vivo* tumorigenicity analysis revealed that animals transplanted with the MUC4 overexpressing cells (AGS-MUC4) had a greater incidence of tumours (83%) in comparison to empty vector control (17%). In addition, the expression of MUC4 resulted in enhanced expression of total cellular ErbB2 and phosphorylated ErbB2. In conclusion, our results showed that MUC4 is overexpressed in gastric adenocarcinoma tissues, and that it has a role in promoting aggressive properties in poorly differentiated gastric non-SRCC cells through the activation of the ErbB2 oncoprotein.

Gastric cancer is the fourth most commonly found cancer worldwide and more than 90% of gastric cancers are adenocarcinomas (Correa *et al*, 2004). According to recent statistical information, in the United States, 21 500 new gastric cancer cases are estimated for the year 2008 (Jemal *et al*, 2008). Despite advances in diagnostic techniques such as imaging, esophagogastroduodenoscopy, magnetic resonance imaging and dual-phase spiral computer tomography, early diagnosis of gastric adenocarcinoma is still a diagnostic problem for clinicians. An early detection with accurate diagnosis and effective surgical or endoscopic treatment can result in a better prognosis.

Gastric adenocarcinoma is classified into intestinal and diffuse type of adenocarcinomas (Lauren, 1965). Morphologically, the intestinal-type gastric adenocarcinoma has well-defined glands with epithelial lining and diffuse-type gastric adenocarcinoma, which mainly consists of scattered individual cells or clusters of cells. It has been shown that the prevalence of poorly differentiated gastric carcinomas is higher than well differentiated gastric cancers (Nakamura *et al*, 1999).

Deregulation of mucins has been shown to be critical in a number of gastro-intestinal malignancies including gastric cancer. Normal gastric epithelial cells express a variety of mucins and have different functions, such as protection against mechanical and infectious insults, lubrication and acid resistance (Tasman-Jones, 1985; Hollingsworth and Swanson, 2004). Various reports have shown that altered mucin carbohydrate and peptide residues of mucins may be used as molecular markers of an increased risk of malignant transformation (Girling *et al*, 1989; Hakomori, 1989; Merlo *et al*, 1989; Ho *et al*, 1993; Springer *et al*, 1995; Llinares *et al*, 2004). Recent studies have provided strong evidences, which potentiate the role of mucins in the pathogenesis of various malignancies (Li *et al*, 2003; Yin *et al*, 2003; Singh *et al*, 2004). In gastric adenocarcinoma, mucin expression pattern is heterogeneous. Mucins in gastric carcinoma include normal mucins of stomach like MUC1, MUC5AC, MUC6 and *de novo* expression of the intestinal mucins MUC2 (Carrato *et al*, 1994; Ho *et al*, 1995; Sakamoto *et al*, 1997; Baldus *et al*, 1998; Reis *et al*, 1998, 2000; Utsunomiya *et al*, 1998).

MUC4, is a membrane-bound mucin and has a significant role in different cancers including pancreatic and breast cancers (Singh *et al*, 2004). MUC4 expression is also associated with the poor prognosis for pancreatic, lung and bile duct cancer patients. Overexpression of MUC4 in pancreatic cancer potentiates pancreatic tumour cell proliferation, survival and invasive properties and also interferes with its interaction to extracellular matrix proteins (Chaturvedi *et al*, 2007). MUC4 also interacts with ErbB2, a growth factor receptor, stabilizes it at the cell surface and hence has an important function in modulating ErbB2-mediated oncogenic signaling in pancreatic cancer cells (Chaturvedi *et al*, 2008). The importance of MUC4 for activation of ErbB2 in poorly differentiated gastric SRCC has been reported (Yokoyama *et al*, 2007). However, to date there is a lack of knowledge about the functional significance of MUC4 in poorly differentiated gastric cancers other than the SRCC subtypes.

Realizing the importance of MUC4 in different malignancies, we prompted to investigate the expression pattern of MUC4 in different gastric adenocarcinomas. In this study, we have shown that MUC4 is overexpressed in the gastric cancer and its expression pattern does not correlate with type, differentiation or stage of cancer. In consideration of MUC4 expression in poorly differentiated gastric non-SRCC cells and its role in the activation of ErbB2 in gastric SRCC cells, we did *in vitro* and *in vivo* studies to check the significance of MUC4 in non-SRCC cells. Here, we have shown that MUC4 overexpression in poorly differentiated AGS, gastric cancer cells, increases its aggressive cancer property in both *in vitro* and *in vivo* experiments. In addition, overexpression of MUC4 in AGS, gastric cancer cells, increases both total and phosphorylated form of ErbB2.

## Materials and methods

### Cell culture and transfection

AGS, MKN45 and KATOIII cell lines were obtained from the American Type Culture Collection (ATCC, Manassas, VA, USA). AGS cells were grown in Ham's F/12 supplemented with 10% fetal bovine serum, MKN45 cells were grown in RPMI-1640 supplemented with 10% fetal bovine serum and KATOIII cells were grown in RPMI-1640 supplemented with 20% fetal bovine serum. In all the media 100 g ml^−1^ penicillin and streptomycin were added. Cell lines were maintained in a 37°C incubator with 5% CO_2_ and in a humidified atmosphere. For the overexpression of MUC4 an engineered MUC4 construct (MUC4minigene) was made, which contains 10% of the tandem repeat sequence. The MUC4minigene construct was stably transfected in AGS cell line for constitutive expression of MUC4. For control, cells were transfected with empty vector (pSecTagC). Stable clones were then selected in a medium containing Zeocin (400 mg ml^−1^). The Zeocin-resistant colonies were isolated by the ring cloning method and maintained in the medium supplemented with Zeocin. The medium was replaced with a complete medium without antibiotic supplement for at least 5 days before any analysis.

### Immunoblot assay

The AGS, MKN45 and KATOIII cell lines were processed for extraction of whole cell protein and using standard protocol western blotting was carried out. The cells were washed two times with phosphate-buffered saline (PBS) and scraped in radioimmunoprecipitation assay buffer (50 mM Tris, 5 mM ethylenediaminetetraacetic acid (EDTA), 150mM NaCl, 0.25% sodium deoxycholate, 1% NP40 pH 7.5), supplemented with protease inhibitor mixture (Roche Diagnostics, Mannheim, Germany) and kept at 4°C for at least 30 min. Cell lysates were passed through the 28G tuberculin needle or alternatively subjected to one freeze thaw cycle to facilitate the disruption of the cell membranes. Cell lysates were centrifuged at 14 000 r.p.m. for 20 min at 4°C and supernatants were collected. Using a BIO-RAD DC protein estimation kit the samples were quantified. Owing to the large size of MUC4, the protein samples were resolved by electrophoresis on a 2% SDS-agarose gel under reducing condition. Resolved proteins were transferred onto the polyvinylidene difluorided membrane. For MUC4 detection anti-MUC4 mouse monoclonal antibody (8G7) was used. For the detection of total ErbB2/HER2 and phosphorylated ErbB2/HER2, anti-HER2 rabbit polyclonal (Santa Cruz Biotechnology, Santa Cruz, CA, USA) and pY1248-HER2 (Upstate Biotech, Lake Placid, NY, USA) antibodies were used respectively. Secondary antibodies consisted of horseradish peroxidase-conjugated anti-mouse/anti-rabbit, which were used for the immunodetection. The blots were processed with ECL chemiluminescence kit (Amersham Biosciences, Piscataway, NJ, USA), and the signal was detected by exposing the processed blots to X-ray films (Biomax Films, Kodak, NY, USA).

### Confocal immunofluorescence microscopy

Cells were grown at low density on sterile coverslips for 20 h. After washing with 0.1 M HEPES containing Hanks buffer, the cells were fixed in ice-cold methanol at −20°C for 2 min. Nonspecific blocking was done by using 10% goat serum containing 0.05% Tween-20 for 30 min, followed by incubation with anti-MUC4 monoclonal antibody (8G7) in PBS for 90 min at room temperature. Cells were washed 3–4 times with PBS containing 0.05% Tween-20 (PBS-T) and then incubated with FITC-conjugated goat anti-mouse secondary antibodies for 60 min. The cells were counterstained with propidium iodide. Finally slides were washed two times with PBS and mounted on glass slides in antifade Vectashield mounting medium (Vector Laboratories, Burlingame, CA, USA). The slides were observed under a ZEISS confocal laser-scanning microscope, and photographs were captured digitally using 510 software.

### Tissue microarrays and immunohistochemistry

To have more number of sample sizes, tissue microarray slides were ordered from two different companies. The AccuMax TM (cat. no. A209) has total of 50 different cancer cases and four non-neoplastic tissues (one corresponding, three non-corresponding). The TMA slide had two spots for each cancer tissue and one spot for each non-neoplastic tissue. Tissue microarray slide from US Biomax Inc. (ST801) had a total of 40 individual cases and each normal adjacent tissue is placed next to its matched cancer tissue. As per recommendations of the manufacturer, before conducting immunohistochemistry, the slides were baked at 60°C for 2 h. Then slides were deparaffinized by using EzDewax (Bio Genex, CA, USA) for 30 min. Sections were hydrated through graded alcohol and endogenous peroxidase activity was quenched by incubating the sections in 0.3% H_2_O_2_ in methanol for 30 min. After washing the slides in PBS (5 min × 2), antigen retrieval was done by heating the slides in citrate buffer (0.01 M, pH 6.0) at 80°C for 20 min. After heating the samples were allowed to cool for 15–20 min in room temperature. This was followed by washing with PBS (5 min × 2). Nonspecific binding was blocked by incubating the sections with 2.5% horse serum for 30 min (Impress reagent Kit,Vector).This was followed by washing with PBS (5 min × 2). Sections were then incubated with anti-MUC4 monoclonal antibody (1 : 2500) at 4°C overnight. Then slides were washed and incubated with secondary antibody (peroxidase labeled Universal anti-mouse/anti-rabbit IgG (Vector, CA)) for 30 min. Then sections were washed with PBS followed by treatment with DAB reagents (0.2 mg ml^−1^) and incubated for 10 min. After washing with distilled water, counter staining was done by using haematoxylin (Vector, CA, USA). After washing in tap water, sections were dehydrated in graded alcohol and after air drying the slides were mounted in permount permanent mounting media (Fisher Scientific, Fair Lawn, NJ, USA). All slides were observed under Nikon E400 light microscope and representative photographs were taken.

### Motility assay

For motility assay, 1 × 10^6^ cells were plated on the top chamber of a non-coated polyethylene teraphthalate membrane (six-well inset, pore size 8 *μ*m; Becton Dickinson, Franklin Lakes, NJ, USA). The bottom chamber contained 1.0 ml DMEM supplemented with 10% fetal bovine serum. The cells were incubated for 24 h, and the cells that did not migrate through the pores in the membrane were removed by scraping the membrane with a cotton swab. Cells that transversed the membrane were stained with Diff-Quick cell staining kit (Dade Behring Inc., Newark, DE, USA). Cells in 10 random fields of view at × 100 magnifications were counted and expressed as the average number of cells/field of view. Three independent experiments were done in each case. These data were represented as the average of the three independent experiments with the s.d. of the average indicated.

### Aggregation assay

Cells were tested for their ability to aggregate in hanging drop suspension cultures. Cells were trypsinized in the presence of EDTA, washed two times in PBS, and resuspended at 2.5 × 10^5^ cells ml^−1^ in the appropriate medium containing 10% fetal bovine serum. Drops (20 *μ*l each) of medium, containing 5000 cells/drop, were pipetted onto the inner surface of the lid of a Petri dish. The lid was then placed on the Petri dish so that the drops were hanging from the lid with the cells suspended within them. To eliminate evaporation, 8 ml of serum-free culture medium were placed in the bottom of the Petri dish. After overnight incubation at 37°C, the lid of the Petri dish was inverted and photographed using a Nikon TS100 inverted tissue culture microscope at × 40 magnifications.

### *In vivo* tumorigenicity assay

To test the tumorigenic capacity, the MUC4-transfected AGS cells along with the control cells were harvested from subconfluent cultures by a brief exposure to 0.25% trypsin and 0.02% EDTA. After neutralising the effect of trypsin with 10% fetal bovine serum, the cells were washed once in PBS. Cell viability and number were determined by trypan blue staining using a hemocytometer. Cells were resuspended in a normal saline solution at a concentration of 25 × 10^6^ cells ml^−1^. Single-cell suspensions of >90% viability was used for the injections. Immunodeficient mice were purchased from the Animal Production Area of the National Cancer Institute-Frederick Cancer Research and Development Center (Frederick, MD, USA). The mice were housed in specific pathogen-free conditions and fed sterile water and food *ad libitum*. The mice were treated in accordance with the Institutional Animal Care and Use Committee guidelines. 5 × 10^6^ viable MUC4-transfected AGS-MUC4 cells, resuspended in a normal saline solution, were injected subcutaneously in six immunodeficient mice. Empty vector transfected cells (AGS-pSecTagC) were used as a control (*n*=6). The animals were monitored two times weekly for tumour formation up to 4 months after inoculation. A palpable mass was observed at the inoculation site at around 80 days of post-injection, followed by rapid growth of the tumour. All mice were killed on day 120 after implantation, and the incidence of tumour was determined.

### Statistical analysis

Subjects with normal or adenocarcinoma or signet ring cell carcinoma were included in the analysis. The distribution of type, grade and stage was compared between positive and negative intensity groups using a *χ*^2^ test or Fisher's Exact test where appropriate. Tumour incidence was compared between groups using Fisher's Exact test.

## Results

### Immunohistochemical analysis of MUC4 expression in gastric cancer tissues

**Figure 1:**
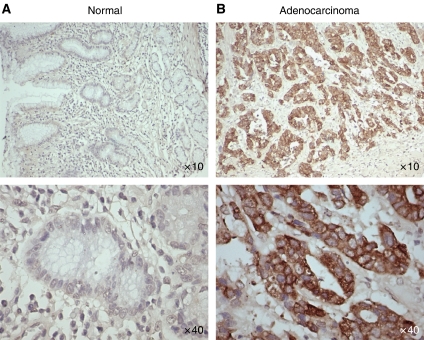
Immunohistochemical analysis of gastric tissues for MUC4 expression by using gastric cancer tissue microarray (TMA) slides. Tissue sections were stained for MUC4 using anti-MUC4 monoclonal antibody followed by biotinylated secondary antibody incubation and streptavidin peroxidase 3,3′-diaminobenzidine-chromogen detection. All the sections were examined under microscope and the immunoreactivity was judged by dark brown staining. (**A**) Representative picture of stained gastric normal adjacent tissues showing no visible MUC4 staining. (**B**) Representative picture of gastric adenocarcinoma tissues showing diffused MUC4 staining. All sections were counter stained with haematoxylin. In all the top panels, original magnification is × 10 and in bottom panels, original magnification is × 40.

As a first step toward studying the role of MUC4 in gastric cancer, we did immunohistochemical analysis of MUC4 expression on TMA samples of gastric adenocarcinoma and normal adjacent area. MUC4 staining showed a diffuse staining pattern (membrane/cytoplasmic) in most of the tissue sections. Representative pictures of the stained gastric tumour tissue sections are presented in Figure 1. Statistical analysis was conducted to determine the association of MUC4 expression pattern with cancer type, differentiation and stage of the tumour (Tables 1 and 2). Out of a total of 128 tissue spots, we found that the proportion of MUC4-positive staining is lower for normal adjacent spots (*n*=45, 9%) compared with patients with adenocarcinoma (*n*=58, 43%) and signet ring cell carcinoma (*n*=25, 32%) (*P*<0.001). However, no significant difference was found in MUC4 expression between adenocarcinomas and signet ring cell carcinomas (*P*=0.34). Furthermore, no significant correlation was found between tumour differentiation and MUC4 expression for all cancer patients combined (*P*=0.34) (Table 1). MUC4 expression pattern did not significantly correlate with the stage of tumour, both individually (*P*=0.16) or in groups, that is, I/II (early stage) and III/IV (late stage) (*P*=0.21) (Table 2).

### Expression of MUC4 in different gastric cancer cell lines

**Figure 2:**
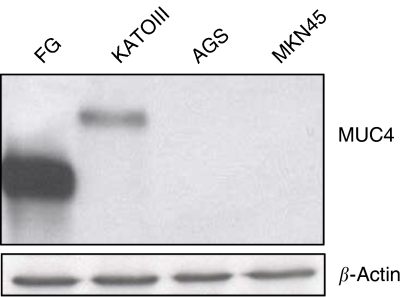
Western blot analysis of MUC4 expression in gastric cancer cell lines. Total protein lysates from AGS, KATOIII and MKN45 gastric cancer cells were prepared. Protein lysate from FG (pancreatic cancer cell line) cells was taken as a positive control. Protein lysates were electrophoretically resolved on 2% agarose gel. Resolved proteins were transferred onto PVDF membrane and probed with MUC4 MAb (8G7) and detected using Amersham HRP-conjugated secondary antibody and ECL kit. Immunoblot of *β*-actin, obtained from 10% SDS–PAGE/Western, was used as an internal control to correct for the loading variation.

**Figure 3:**
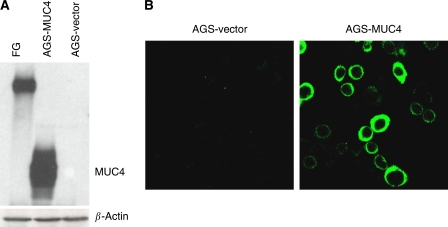
Expression of MUC4 in AGS and its derived sublines: AGS-vector (empty vector transfected) and AGS-MUC4 (MUC4-transfected) cells. (**A**) Western blot analysis: total protein lysates were prepared from the subconfluent cells. A total of 20 *μ*g protein from cell extracts was electrophoretically resolved on 2% Agarose gel. Resolved proteins were transferred onto PVDF membrane and probed with MUC4 MAb (8G7). Protein from FG (pancreatic cancer cell line) cells was taken as a positive control. (**B**) Expression analysis of MUC4 using confocal microscopy: Cells were grown at a low density on sterilised cover slips; after methanol fixation, slides were incubated with MUC4 MAb (8G7), followed by FITC-conjugated secondary antibody, and were observed under a ZEISS confocal laser-scanning microscope (magnification, × 630).

Using immunoblot assay, MUC4 expression was checked in three different types of gastric adenocarcinoma cell lines (AGS, MKN45 and KATOIII). MUC4 expression was detected in KATOIII (SRCC) but was undetectable in AGS and MKN45 cell lines (Figure 2). To further analyse the role of MUC4 in the aggressiveness of non-signet ring cell type poorly differentiated gastric adenocarcinomas, MUC4 was ectopically expressed in AGS gastric cancer cell line (using an engineered MUC4 cDNA construct, MUC4minigene (Moniaux *et al*, 2007)). The MUC4minigene's deduced protein (320 kDa) is analogous to that of wild-type MUC4 protein (930 kDa) (Moniaux *et al*, 2007). Western blot analysis (Figure 3) and confocal study showed the overexpression of MUC4 in selected clones transfected with MUC4 construct in comparison to empty vector transfected clones (Figure 3). Furthermore, confocal analysis showed the membrane localisation of MUC4 in AGS-MUC4 clones.

### MUC4 overexpression increases cell motility in AGS gastric cancer cells

**Figure 4:**
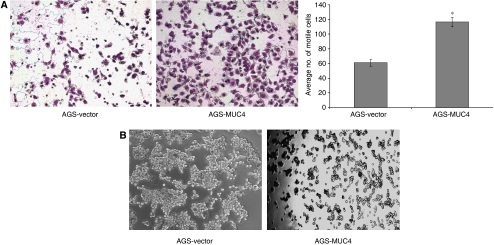
Phenotypic changes of AGS-MUC4 cells compared with AGS-vector cells. (**A**) Cell motility assay: MUC4 expression correlates with the cell motility. Cells (1 × 10^6^) were plated in the top chamber of noncoated polyethylene teraphthalate membranes and incubated for 20 h. Cells that transversed the membranes were stained with a Diff-Quick cell staining kit. The number of cells transversing the membrane was determined by averaging 10 random fields of view at × 100 and expressed as the average number of cells/field of view and is the average of two independent experiments. Mean±s.e.; *n*=20; ^*^P<0.005. Cell motility was significantly (*P*<0.005) increased in MUC4-transfected AGS cells. (**B**) Aggregation assay: drops of medium (20 *μ*l each) containing 500 cells/drop were pipetted onto the inner surface of the lid of a Petri dish. After overnight incubation at 37°C, the lid of the Petri dish was inverted and photographed using a Nikon TS100 inverted tissue culture microscope at × 40 magnification. An increased cellular aggregation observed in AGS-MUC4 cells.

The aggressiveness of a malignant cell depends on its potential to invade the ECM and its ability to metastasize to distant sites. Different studies have shown that the invasive and metastasis potential of cancer cells are strongly related to a variety of phenotypic characteristics. Among these characteristics, motility of cells highly influences the metastatic property of cells (Yamaguchi *et al*, 2005). As the MUC4 overexpression is associated with an increased motility of pancreatic cancer cells, we examined whether the MUC4 overexpression in gastric cancer is associated with an increase in cell motility or not. Cell motility was determined on top of the uncoated porous membrane. The number of MUC4 overexpressing AGS gastric cancer cells (AGS-MUC4) migrated to the lower surface of the porous membrane was significantly high (*P*<0.005) than that of the vector control (AGS-vector) cells (Figure 4A).

### Overexpression of MUC4 decreases aggregation property of AGS gastric cancer cells

Like cell motility, aggregation property of cells is also a critical factor, which affects the metastasis property of tumour cells. The aggregation property is usually deregulated in the tumour cells because of alteration in the expression of different cell surface molecules (Sommers, 1996; Komatsu *et al*, 1997; Truant *et al*, 2003). Therefore, to test the effect of MUC4 overexpression on adhesiveness of AGS cells, we used the aggregation assay described in Materials and methods. MUC4 overexpression showed a decrease in aggregation of AGS-MUC4-transfected cells in comparison to vector-transfected control cells (Figure 4B).

### Overexpression of MUC4 enhances tumorigenicity of AGS gastric cancer cells in nude mice

To investigate the role of MUC4 on tumorigenic property of gastric cancer cells, AGS-MUC4 and AGS-vector cells, were injected subcutaneously into immune deficient nude mice. 5 × 10^6^ cells were injected and a palpable mass was first noticed at the eightieth day of post-inoculation and continued to grow up to 120 days. Among the six mice inoculated with AGS-MUC4 cells, incidence of tumour was observed in five mice (83%), whereas only one mouse had a tumour among the six mice inoculated with AGS-vector cells (17%). Further, statistical analysis showed that this difference in the incidence of the tumour between the two groups is marginally significant (*P*=0.08) (Table 3).

### Overexpression of MUC4 activates ErbB2 oncoprotein in AGS gastric cancer cells

**Figure 5:**
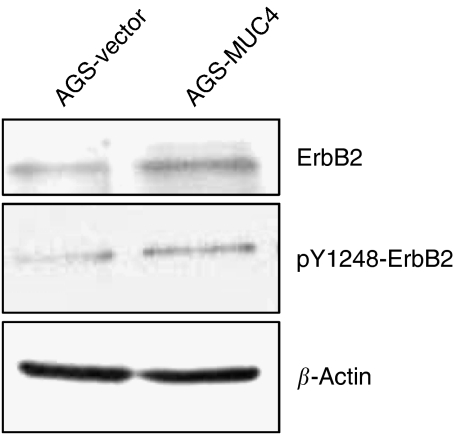
Effect of MUC4 expression on ErbB2 expression and phosphorylation. (**A**) Western blot analysis: a total of 20 *μ*g of protein from AGS-derived cell lines, were resolved by SDS-PAGE and transferred to PVDF membrane and probed with antibodies against, ErbB2, Phospho-tyr 1248 ErbB2, and *β*-actin. MUC4-transfected AGS cells showed an increased level of total and phosphorylated ErbB2 in comparison to vector-transfected cells.

shRNA-mediated knock-down of MUC4 in pancreatic cancer cells showed a decrease in the total levels and phosphorylated form of ErbB2 protein (at Tyr^1248^) and was shown that overexpression of MUC4 plays a crucial role in stabilising ErbB2 (Chaturvedi *et al*, 2007, 2008). Other studies have shown that in SRCC type gastric cancer cells, MUC4 is required for the activation of ErbB2 (Yokoyama *et al*, 2007). On the basis of these earlier observations, we wanted to determine whether MUC4 exerts its function through regulating ErbB2 expression and activation in AGS-MUC4 gastric cancer cells. The expression of total and active form of ErbB2 was measured by western blot analysis. Cell lysate of AGS-MUC4 showed increased level of total and phosphorylated (at Tyr^1248^) ErbB2 protein compared with the control AGS-vector cell lysate (Figure 5).

## Discussion

MUC4 is one of the most widely studied membrane-bound mucins having a significant function in the pathogenesis of several cancers (Shibahara *et al*, 2004; Singh *et al*, 2004). The aberrant expression of MUC4 has been reported in different types of carcinomas (Andrianifahanana *et al*, 2001; Llinares *et al*, 2004; Weed *et al*, 2004). It has been shown that MUC4 expression correlates with cancer progression (Swartz *et al*, 2002; Park *et al*, 2003). In this study, we showed that MUC4 is overexpressed in gastric cancer tissues as compared with normal adjacent tissues. Overexpression of MUC4 was associated with an aggressive phenotype of gastric cancer cells. MUC4 overexpression also increased the activation of ErbB2 oncoprotein. Hence, our study provides for the first time, the importance of MUC4 in gastric cancer and explains the possible mechanism through which MUC4 can promote aggressive property of poorly differentiated gastric non-SRCC cells.

In this study, we examined and compared the expression of MUC4 in gastric cancer tissues. MUC4 showed a significant overexpression in gastric cancer tissues compared with the normal adjacent tissues. Similar findings showing the overexpression of MUC4 in gastric adenocarcinoma has been reported earlier (Lopez-Ferrer *et al*, 2000). MUC4 is known to be expressed in the embryonic gastric tissues around 8 weeks of gestation (Buisine *et al*, 2000). It has also been shown that many embryogenesis phenomena like cell proliferation, lineage allocation, cell migration and differentiation of cells are also seen during cancer progression. Therefore, overexpression of MUC4 in adult gastric carcinoma supports the concept of ‘fetal antigen’ expression during malignant condition and indicates its possible role in gastric cancer progression. Further, our statistical analysis showed that there is no significant association between MUC4 expression with type, grade of differentiation and stage of gastric cancer. This suggests that MUC4 itself may not be a potential marker for early diagnosis of gastric cancer. Previous studies performed regarding the role of mucins as diagnostic and prognosis markers in gastric carcinoma tissue showed contradictory results (Correa and Shiao, 1994; Byrd *et al*, 1997). Therefore, for higher accuracy, many studies have been done to evaluate the combined expression pattern of mucins with other molecules such as E-cadherin with MUC1 expression (Tanaka *et al*, 2003). Similarly, comparing the expression pattern of MUC4 with other proteins like E-cadherin or other mucins will strengthen the study and may potentiate the possible use of MUC4 as a diagnostic and prognostic marker for gastric adenocarcinomas.

Different studies have shown the functional role of MUC4 in tumorigenicity and metastasis property of cancer cells (Singh *et al*, 2004; Chaturvedi *et al*, 2007). Recently, we have also shown that in pancreatic cancer cells MUC4 interacts with ErbB2 and stabilizes its localization on the cell membrane (Chaturvedi *et al*, 2008). In another very recent study, it has been shown that MUC4 interacts with ErbB2 in human gallbladder carcinoma and helps in the activation of erbB2 (Miyahara *et al*, 2008).Till date, information regarding the role of MUC4 in gastric cancer is very less, recently, it has been shown that specifically in poorly differentiated type gastric signet ring cell carcinoma cells, MUC4 is required for the activation of ErbB2. Because MUC4 is expressed even in poorly differentiated gastric non-SRCC cells, we therefore reasoned that MUC4 might have a possible role in those kinds of cells. Here, we report that out of the three poorly differentiated cells (KATOIII, MKN45 and AGS), only KATOIII, which is a signet ring cell carcinoma cell line, expresses MUC4.

Further, to check the actual role of MUC4 in non-SRCC type poorly differentiated gastric cancer cells, MUC4 was ectopically overexpressed in a gastric adenocarcinoma cell line (AGS), which has an undetectable expression level of MUC4. In different *in vitro* studies, we found that, MUC4 causes an increase in the motility of AGS-MUC4 cells, and a decrease in the adhesive property of AGS-MUC4 cells. This finding supports other studies where MUC4 has a similar function in other cancers, such as pancreatic cancer (Singh *et al*, 2004; Chaturvedi *et al*, 2007). The increase in cell motility (*P*<0.05), which has a major role during the dynamic process of tumour invasion and metastasis, may be a direct result of MUC4-mediated changes in the actin organisation, or indirectly through an ErbB2-mediated pathway (Singh *et al*, 2007). During cancer cell metastasis, cells remain loosely attached to the ECM or to the other cells. This property is essential to make the cells more migratory and to increase the invasiveness of cancer cells. In our aggregation assay, we found that MUC4 overexpression in AGS cell line, decreases its aggregation property, or in other words enhances its metastatic property. This decreased adhesive property among cells may be because of charge–charge repulsion on account of the presence of negatively charged O-glycosidic chains present in the central repetitive domain of MUC4 or owing to disruption of integrin-mediated cell adhesion. Furthermore, the high incidence of tumours in animals injected with AGS-MUC4 cells than animals inoculated with AGS-vector cells, indicate the role of MUC4 in tumorigenicity of gastric cancer cells.

Decrease in cell death and increase in cell proliferation are two major regulatory elements in enhancing the tumorigenicity of cancer cells. Overexpression of ErbB2 oncoprotein has been shown to correlate with tumour aggressiveness in various tumours (Hynes and Lane, 2005). In gastric cancer, a correlation between ErbB2 gene amplification and prognosis of patients has been reported (Nakajima *et al*, 1999; Lin *et al*, 2000). In this study, similar to our previous report, we also observed an increase in total ErbB2 and phosphorylated ErbB2 expression in MUC4 expressing AGS cells. AGS, is a well studied poorly differentiated cell line (Barranco *et al*, 1983). The tumorigenicity of this cell line in animals has been already reported (Barranco *et al*, 1983). Here, the increase in tumorigenicity of AGS cells mediated by MUC4 may be on account of the interaction of MUC4 with ErbB2 and further stabilisation and activation of ErbB2-mediated oncogenic signaling.

In conclusion, our *in vitro* and *in vivo* studies showed a significant role of MUC4 in promoting the aggressiveness and tumorigenicity of poorly differentiated gastric non-SRCC cells. In addition, we showed a possible mechanism through which MUC4 can increase the tumorigenicity property of poorly differentiated gastric non-SRCC cells. Validation for MUC4 expression as a prognostic marker requires further studies. A study on the combined expression pattern of MUC4 and other molecules like E-cadherin or other mucins may provide more accuracy and specificity to use MUC4 as a diagnostic or prognostic marker for gastric cancer. Our study also indicates that MUC4 can be targeted for treatment of gastric cancer.

## Figures and Tables

**Table 1:** Association of MUC4 expression pattern with types and grades of gastric cancer

**Tissues**	**MUC4-negative *N* (%)**	**MUC4-positive *N* (%)**	***P*-value**
*Types*			
Normal	41 (91%)	4 (9%)	*P*<0.001
Adenocarcinoma	33 (57%)	25 (43%)	
SRCC	17 (68%)	8 (32%)	
			
*Differentiation*			
Moderate	9 (53%)	8 (47%)	
Poor	30 (58%)	22 (42%)	*P*=0.34
Not	4 (100%)	0 (0%)	
Well	7 (70%)	3 (30%)	

**Table 2:** Association of MUC4 expression pattern with stages of gastric cancer

**Cancer stage**	**MUC4-negative *N*=28**	**MUC4-positive *N*=19**	***P*-value**
I	11 (58%)	8 (42%)	0.16^***^
II	6 (100%)	0 (0%)	
III	9 (50%)	9 (50%)	
IV	2 (50%)	2 (50%)	

^***^Fisher's Exact test.

Information regarding the tumour stage was not available for all the spots.

**Table 3:** Incidence of tumours in animals injected with AGS-MUC4 and AGS-pSecTagC cells

**Name of the group**	**Number of animals injected**	**Number of animals having tumour**	***P*-value**
AGS-MUC4	6	5 (83%)	*P*=0.08^***^
AGS-Vector	6	1 (17%)	

^***^ Fisher's Exact test.

## References

[bib1] Andrianifahanana M, Moniaux N, Schmied BM, Ringel J, Friess H, Hollingsworth MA, Buchler MW, Aubert JP, Batra SK (2001) Mucin (MUC) gene expression in human pancreatic adenocarcinoma and chronic pancreatitis: a potential role of MUC4 as a tumor marker of diagnostic significance. Clin Cancer Res 7: 4033–4040 11751498

[bib2] Baldus SE, Zirbes TK, Engel S, Hanisch FG, Monig SP, Lorenzen J, Glossmann J, Fromm S, Thiele J, Pichlmaier H, Dienes HP (1998) Correlation of the immunohistochemical reactivity of mucin peptide cores MUC1 and MUC2 with the histopathological subtype and prognosis of gastric carcinomas. Int J Cancer 79: 133–138 9583726 10.1002/(sici)1097-0215(19980417)79:2<133::aid-ijc6>3.0.co;2-u

[bib3] Barranco SC, Townsend Jr CM, Casartelli C, Macik BG, Burger NL, Boerwinkle WR, Gourley WK (1983) Establishment and characterization of an *in vitro* model system for human adenocarcinoma of the stomach. Cancer Res 43: 1703–1709 6831414

[bib4] Buisine MP, Devisme L, Maunoury V, Deschodt E, Gosselin B, Copin MC, Aubert JP, Porchet N (2000) Developmental mucin gene expression in the gastroduodenal tract and accessory digestive glands. I. Stomach. A relationship to gastric carcinoma. J Histochem Cytochem 48: 1657–1666 11101634 10.1177/002215540004801209

[bib5] Byrd JC, Yan P, Sternberg L, Yunker CK, Scheiman JM, Bresalier RS (1997) Aberrant expression of gland-type gastric mucin in the surface epithelium of Helicobacter pylori-infected patients. Gastroenterology 113: 455–464 9247464 10.1053/gast.1997.v113.pm9247464

[bib6] Carrato C, Balague C, De BC, Gonzalez E, Gambus G, Planas J, Perini JM, Andreu D, Real FX (1994) Differential apomucin expression in normal and neoplastic human gastrointestinal tissues. Gastroenterology 107: 160–172 8020658 10.1016/0016-5085(94)90073-6

[bib7] Chaturvedi P, Singh AP, Chakraborty S, Chauhan SC, Bafna S, Meza JL, Singh PK, Hollingsworth MA, Mehta PP, Batra SK (2008) MUC4 mucin interacts with and stabilizes the HER2 oncoprotein in human pancreatic cancer cells. Cancer Res 68: 2065–2070 18381409 10.1158/0008-5472.CAN-07-6041 PMC2835497

[bib8] Chaturvedi P, Singh AP, Moniaux N, Senapati S, Chakraborty S, Meza JL, Batra SK (2007) MUC4 mucin potentiates pancreatic tumor cell proliferation, survival, and invasive properties and interferes with its interaction to extracellular matrix proteins. Mol Cancer Res 5: 309–320 17406026 10.1158/1541-7786.MCR-06-0353

[bib9] Correa P, Shiao YH (1994) Phenotypic and genotypic events in gastric carcinogenesis. Cancer Res 54: 1941s–1943s 8137316

[bib10] Correa P, Piazuelo MB, Camargo MC (2004) The future of gastric cancer prevention. Gastric Cancer 7: 9–16 15052434 10.1007/s10120-003-0265-0

[bib11] Girling A, Bartkova J, Burchell J, Gendler S, Gillett C, Taylor-Papadimitriou J (1989) A core protein epitope of the polymorphic epithelial mucin detected by the monoclonal antibody SM-3 is selectively exposed in a range of primary carcinomas. Int J Cancer 43: 1072–1076 2471698 10.1002/ijc.2910430620

[bib12] Hakomori S (1989) Aberrant glycosylation in tumors and tumor-associated carbohydrate antigens. Adv Cancer Res 52: 257–331 2662714 10.1016/s0065-230x(08)60215-8

[bib13] Ho SB, Niehans GA, Lyftogt C, Yan PS, Cherwitz DL, Gum ET, Dahiya R, Kim YS (1993) Heterogeneity of mucin gene expression in normal and neoplastic tissues. Cancer Res 53: 641–651 7678777

[bib14] Ho SB, Shekels LL, Toribara NW, Kim YS, Lyftogt C, Cherwitz DL, Niehans GA (1995) Mucin gene expression in normal, preneoplastic, and neoplastic human gastric epithelium. Cancer Res 55: 2681–2690 7780985

[bib15] Hollingsworth MA, Swanson BJ (2004) Mucins in cancer: protection and control of the cell surface. Nat Rev Cancer 4: 45–60 14681689 10.1038/nrc1251

[bib16] Hynes NE, Lane HA (2005) ERBB receptors and cancer: the complexity of targeted inhibitors. Nat Rev Cancer 5: 341–354 15864276 10.1038/nrc1609

[bib17] Jemal A, Siegel R, Ward E, Hao Y, Xu J, Murray T, Thun MJ (2008) Cancer statistics, 2008. CA Cancer J Clin 58: 71–96 18287387 10.3322/CA.2007.0010

[bib18] Komatsu M, Carraway CA, Fregien NL, Carraway KL (1997) Reversible disruption of cell-matrix and cell-cell interactions by overexpression of sialomucin complex. J Biol Chem 272: 33245–33254 9407114 10.1074/jbc.272.52.33245

[bib19] Lauren P (1965) The two histological main types of gastric carcinoma: diffuse and so-called intestinal-type carcinoma. an attempt at a histo-clinical classification. Acta Pathol Microbiol Scand 64: 31–49 14320675 10.1111/apm.1965.64.1.31

[bib20] Li Y, Liu D, Chen D, Kharbanda S, Kufe D (2003) Human DF3/MUC1 carcinoma-associated protein functions as an oncogene. Oncogene 22: 6107–6110 12955090 10.1038/sj.onc.1206732 PMC4209839

[bib21] Lin W, Kao HW, Robinson D, Kung HJ, Wu CW, Chen HC (2000) Tyrosine kinases and gastric cancer. Oncogene 19: 5680–5689 11114748 10.1038/sj.onc.1203924

[bib22] Llinares K, Escande F, Aubert S, Buisine MP, De BC, Batra SK, Gosselin B, Aubert JP, Porchet N, Copin MC (2004) Diagnostic value of MUC4 immunostaining in distinguishing epithelial mesothelioma and lung adenocarcinoma. Mod Pathol 17: 150–157 14657954 10.1038/modpathol.3800027

[bib23] Lopez-Ferrer A, De BC, Barranco C, Garrido M, Isern J, Carlstedt I, Reis CA, Torrado J, Real FX (2000) Role of fucosyltransferases in the association between apomucin and Lewis antigen expression in normal and malignant gastric epithelium. Gut 47: 349–356 10940270 10.1136/gut.47.3.349 PMC1728024

[bib24] Merlo GR, Siddiqui J, Cropp CS, Liscia DS, Lidereau R, Callahan R, Kufe DW (1989) Frequent alteration of the DF3 tumor-associated antigen gene in primary human breast carcinomas. Cancer Res 49: 6966–6971 2582438

[bib25] Miyahara N, Shoda J, Ishige K, Kawamoto T, Ueda T, Taki R, Ohkohchi N, Hyodo I, Thomas MB, Krishnamurthy S, Carraway KL, Irimura T (2008) MUC4 interacts with ErbB2 in human gallbladder carcinoma: potential pathobiological implications. Eur J Cancer 44: 1048–1056 18397823 10.1016/j.ejca.2008.03.007

[bib26] Moniaux N, Chaturvedi P, Varshney GC, Meza JL, Rodriguez-Sierra JF, Aubert JP, Batra SK (2007) Human MUC4 mucin induces ultra-structural changes and tumorigenicity in pancreatic cancer cells. Br J Cancer 97: 345–357 17595659 10.1038/sj.bjc.6603868 PMC2360313

[bib27] Nakajima M, Sawada H, Yamada Y, Watanabe A, Tatsumi M, Yamashita J, Matsuda M, Sakaguchi T, Hirao T, Nakano H (1999) The prognostic significance of amplification and overexpression of c-met and c-erb B-2 in human gastric carcinomas. Cancer 85: 1894–1902 10223227 10.1002/(sici)1097-0142(19990501)85:9<1894::aid-cncr3>3.0.co;2-j

[bib28] Nakamura T, Yao T, Niho Y, Tsuneyoshi M (1999) A clinicopathological study in young patients with gastric carcinoma. J Surg Oncol 71: 214–219 10440758 10.1002/(sici)1096-9098(199908)71:4<214::aid-jso2>3.0.co;2-d

[bib29] Park HU, Kim JW, Kim GE, Bae HI, Crawley SC, Yang SC, Gum Jr JR, Batra SK, Rousseau K, Swallow DM, Sleisenger MH, Kim YS (2003) Aberrant expression of MUC3 and MUC4 membrane-associated mucins and sialyl Le(x) antigen in pancreatic intraepithelial neoplasia. Pancreas 26: e48–e54 12657964 10.1097/00006676-200304000-00022

[bib30] Reis CA, David L, Carvalho F, Mandel U, De BC, Mirgorodskaya E, Clausen H, Sobrinho-Simoes M (2000) Immunohistochemical study of the expression of MUC6 mucin and co-expression of other secreted mucins (MUC5AC and MUC2) in human gastric carcinomas. J Histochem Cytochem 48: 377–388 10681391 10.1177/002215540004800307

[bib31] Reis CA, David L, Seixas M, Burchell J, Sobrinho-Simoes M (1998) Expression of fully and under-glycosylated forms of MUC1 mucin in gastric carcinoma. Int J Cancer 79: 402–410 9699534 10.1002/(sici)1097-0215(19980821)79:4<402::aid-ijc16>3.0.co;2-6

[bib32] Sakamoto H, Yonezawa S, Utsunomiya T, Tanaka S, Kim YS, Sato E (1997) Mucin antigen expression in gastric carcinomas of young and old adults. Hum Pathol 28: 1056–1065 9308730 10.1016/s0046-8177(97)90059-9

[bib33] Shibahara H, Tamada S, Higashi M, Goto M, Batra SK, Hollingsworth MA, Imai K, Yonezawa S (2004) MUC4 is a novel prognostic factor of intrahepatic cholangiocarcinoma-mass forming type. Hepatology 39: 220–229 14752841 10.1002/hep.20031

[bib34] Singh AP, Chaturvedi P, Batra SK (2007) Emerging roles of MUC4 in cancer: a novel target for diagnosis and therapy. Cancer Res 67: 433–436 17234748 10.1158/0008-5472.CAN-06-3114

[bib35] Singh AP, Moniaux N, Chauhan SC, Meza JL, Batra SK (2004) Inhibition of MUC4 expression suppresses pancreatic tumor cell growth and metastasis. Cancer Res 64: 622–630 14744777 10.1158/0008-5472.can-03-2636

[bib36] Sommers CL (1996) The role of cadherin-mediated adhesion in breast cancer. J Mammary Gland Biol Neoplasia 1: 219–229 10887495 10.1007/BF02013645

[bib37] Springer GF, Desai PR, Ghazizadeh M, Tegtmeyer H (1995) T/Tn pancarcinoma autoantigens: fundamental, diagnostic, and prognostic aspects. Cancer Detect Prev 19: 173–182 7750105

[bib38] Swartz MJ, Batra SK, Varshney GC, Hollingsworth MA, Yeo CJ, Cameron JL, Wilentz RE, Hruban RH, Argani P (2002) MUC4 expression increases progressively in pancreatic intraepithelial neoplasia. Am J Clin Pathol 117: 791–796 12090430 10.1309/7Y7N-M1WM-R0YK-M2VA

[bib39] Tanaka M, Kitajima Y, Sato S, Miyazaki K (2003) Combined evaluation of mucin antigen and E-cadherin expression may help select patients with gastric cancer suitable for minimally invasive therapy. Br J Surg 90: 95–101 12520583 10.1002/bjs.4014

[bib40] Tasman-Jones C (1985) Gastric mucus--physical properties in cytoprotection. Med J Aust 142: S5–S6 10.5694/j.1326-5377.1985.tb128339.x 3969029

[bib41] Truant S, Bruyneel E, Gouyer V, De WO, Pruvot FR, Mareel M, Huet G (2003) Requirement of both mucins and proteoglycans in cell-cell dissociation and invasiveness of colon carcinoma HT-29 cells. Int J Cancer 104: 683–694 12640674 10.1002/ijc.11011

[bib42] Utsunomiya T, Yonezawa S, Sakamoto H, Kitamura H, Hokita S, Aiko T, Tanaka S, Irimura T, Kim YS, Sato E (1998) Expression of MUC1 and MUC2 mucins in gastric carcinomas: its relationship with the prognosis of the patients. Clin Cancer Res 4: 2605–2614 9829723

[bib43] Weed DT, Gomez-Fernandez C, Yasin M, Hamilton-Nelson K, Rodriguez M, Zhang J, Carraway KL (2004) MUC4 and ErbB2 expression in squamous cell carcinoma of the upper aerodigestive tract: correlation with clinical outcomes. Laryngoscope 114: 1–32 10.1097/00005537-200408001-00001 15284539

[bib44] Yamaguchi H, Wyckoff J, Condeelis J (2005) Cell migration in tumors. Curr Opin Cell Biol 17: 559–564 16098726 10.1016/j.ceb.2005.08.002

[bib45] Yin L, Li Y, Ren J, Kuwahara H, Kufe D (2003) Human MUC1 carcinoma antigen regulates intracellular oxidant levels and the apoptotic response to oxidative stress. J Biol Chem 278: 35458–35464 12826677 10.1074/jbc.M301987200

[bib46] Yokoyama A, Shi BH, Kawai T, Konishi H, Andoh R, Tachikawa H, Ihara S, Fukui Y (2007) Muc4 is required for activation of ErbB2 in signet ring carcinoma cell lines. Biochem Biophys Res Commun 355: 200–203 17292332 10.1016/j.bbrc.2007.01.133

